# Viral microRNA effects on persistent infection of human lymphoid cells by polyomavirus SV40

**DOI:** 10.1371/journal.pone.0192799

**Published:** 2018-02-12

**Authors:** Adrienne L. McNees, Lindsay J. Harrigal, Aoife Kelly, Charles G. Minard, Connie Wong, Janet S. Butel

**Affiliations:** 1 Department of Molecular Virology and Microbiology, Baylor College of Medicine, Houston, Texas, United States of America; 2 Institute for Clinical and Translational Research, Baylor College of Medicine, Houston, Texas, United States of America; University of St Andrews, UNITED KINGDOM

## Abstract

**Background:**

Polyomaviruses, including simian virus 40 (SV40), display evidence of lymphotropic properties. This study analyzed the nature of SV40–human lymphocyte interactions in established cell lines and in primary lymphocytes. The effects of viral microRNA and the structure of the viral regulatory region on SV40 persistence were examined.

**Results:**

SV40 DNA was maintained in infected B cell and myeloid cell lines during cell growth for at least 28 days. Limiting dilution analysis showed that low amounts of SV40 DNA (~2 copies per cell) were retained over time. Infected B cells remained viable and able to proliferate. Genome copies of the SV40 microRNA-null mutant persisted at higher levels than the DNA of wild-type viruses. Complex viral regulatory regions produced modestly higher DNA levels than simple regulatory regions. Viral large T-antigen protein was detected at low frequency and at low levels in infected B cells. Following infection of primary lymphocytes, SV40 DNA was detected in CD19^+^ B cells and CD14^+^ monocytes, but not in CD3^+^ T cells. Rescue attempts using either lysates of SV40-infected B lymphocytes, coculture of live cells, or infectious center assays all showed that replication-competent SV40 could be recovered on rare occasions. SV40 infections altered the expression of several B cell surface markers, with more pronounced changes following infections with the microRNA-null mutant.

**Conclusion:**

These findings indicate that SV40 can establish persistent infections in human B lymphocytes. The cells retain low copy numbers of viral DNA; the infections are nonproductive and noncytolytic but can occasionally produce infectious virus. SV40 microRNA negatively regulates the degree of viral effects on B cells.

**Significance:**

Lymphocytes may serve as viral reservoirs and may function to disseminate polyomaviruses to different tissues in a host. To our knowledge, this report is the first extensive analysis of viral microRNA effects on SV40 infection of human lymphocytes.

## Introduction

The polyomavirus family is rapidly expanding [1,2]. However, the pathogenesis of infections by polyomaviruses in susceptible hosts and how those infections may lead to disease (usually in the immunocompromised) are not well-understood. Polyomaviruses are known to establish persistent infections in hosts, but the breadth of target tissues and the status of virus in those tissues remain obscure [3]. Insights into the nature of viral infection and persistence in different cell types are needed.

Lymphocytes are important factors in virus–host interactions for multiple virus families with the precise nature of those interactions differing among virus types. Evidence suggests that polyomaviruses possess lymphotropic properties. Detections of human isolates JC virus (JCV) and BK virus (BKV) in human lymphocytes have been reported for over a decade, including in cells from healthy individuals and from patients with immune deficiencies or progressive multifocal leukoencephalopathy [4–14]. Newer human polyomavirus isolates, MCPyV, KIPyV, WUPyV, TSPyV, HPyV6, HPyV7, MWPyV, and STLPyV also appear to have lymphotropic properties based on detection of viral DNA in lymphoid tissues [15–24], as do lymphotropic papovavirus, LPV, and murine polyoma virus, MuPyV [25–27].

Polyomavirus simian virus 40 (SV40) of rhesus macaque origin is one of the most well-characterized members of the family and the most readily amenable to laboratory studies. Like human polyomaviruses BKV and JCV, SV40 causes a low-grade persistent infection in kidneys in its natural host and shares evidence of lymphotropism. In monkeys infected with simian immunodeficiency virus, SV40 coinfection becomes widespread with virus detected in the brain, lung, kidney, lymph node, spleen and peripheral blood [28–30]. This dissemination likely occurs via hematogenous spread of the virus. SV40 can infect human cells in culture and SV40 DNA has been detected in tonsils and peripheral blood lymphocytes of healthy human donors [31–39].

The goal of this study was to characterize the nature of interactions between polyomavirus SV40 and human lymphoid cells. Specific objectives included the following: (i) to establish the effects of SV40 microRNA (miRNA) and the structure of the viral regulatory region (RR) on patterns of infection of human lymphocytes, (ii) to identify levels of viral DNA and gene expression in persistently infected cells; and (iii) to determine the effects of viral infections on lymphoid cell properties. We found that SV40 establishes chronic, nonproductive infections in B lymphocytes and in myeloid cells that can occasionally yield infectious virus. This could provide a mechanism for viral retention and dissemination throughout the host and contribute to viral pathogenesis and disease. This SV40 system provides a model for studies of the growing number of newly detected polyomaviruses.

## Materials and methods

### Cell lines

Human lymphocyte cell line DG75 (ATCC CRL-2625), derived from an Epstein-Barr virus (EBV)-negative primary abdominal B cell lymphoma [40], was obtained from Paul D. Ling (Baylor College of Medicine). Cell lines BJAB (DSMZ ACC-757), an EBV-negative B-lymphoblastoid cell line [41], and CEM (ATCC CCL-119), developed from an acute T cell leukemia [42], were obtained from Linda R. Gooding (Emory University, Atlanta, GA). Human B lymphocyte cell line RL (ATCC CRL-2261), derived from a non-Hodgkin’s lymphoma ascites [43], and TF-1a (ATCC CRL-2451), a myeloid leukemia cell line that can undergo macrophage-like differentiation [44], were obtained from the ATCC. Lymphocyte and leukemia cell lines were grown in RPMI medium (Cellgro, Manassas, VA) supplemented with 10% fetal bovine serum (FBS; Hyclone, Logan, UT) and 2 mM glutamine. The TC7 cell line, an African green monkey kidney cell line derived from CV-1 cells that is permissive for SV40 replication [45,46], was cultured in Gibco Eagle’s minimum essential medium (Thermo Fisher Scientific, Waltham, MA) supplemented with 5% FBS, 10% tryptose phosphate broth, 2% Gibco MEM vitamin solution, and 0.25% glucose [47].

### Viruses

SV40 virus strains 776 variants 1E and 2E (GenBank accession number J02400), Baylor SVB2E (GenBank accession number AF155358), and VA45-54-2E (GenBank accession number AF156105) have been described [48]. Strain SV40 776-2E-SM1, obtained from Christopher S. Sullivan (University of Texas, Austin, TX), fails to express SV40 miRNA [49,50]. SV40 viral stocks were prepared in TC7 cells and infectious virus titers were quantified by plaque assay [47].

### SV40 infections

Cells of lymphocyte and leukemia cell lines were washed and resuspended at a density of 10^7^ cells/ml in serum-free RPMI media. Virus was added at a multiplicity of infection (MOI) of 5 plaque-forming units (PFU)/cell and allowed to adsorb for 90 min at 37°C. (As particle-to-PFU ratios vary among virus stocks, cultures were exposed to different numbers of viral genomes.) Mock-infected samples were treated following the same protocol, but with serum-free media in place of virus. Cells were washed three times with RPMI containing 10% FBS and then incubated at 10^6^ cells/ml in the same media. Cell samples were harvested at various days postinfection (d.p.i.) for analyses. Cell culture density was adjusted to 10^6^ cells/ml when cells were subcultured every 2 to 4 days.

Primary peripheral blood lymphocytes (PBLs) were isolated by Ficoll Hypaque gradient centrifugation (Sigma) from buffy coats obtained from normal donors at the Texas Gulf Coast Regional Blood Center at different times. Cells were infected with wild-type (WT) SV40 and cultured in RPMI–10% FBS media. Subpopulations of infected cells positive for markers CD19 (B cells), CD3 (T cells), or CD14 (monocytes/macrophages) were isolated using Miltenyi magnetic bead reagents following the manufacturer’s instructions. In some experiments primary lymphocytes were activated by treatment with 1 μg/ml of lipopolysaccharide (LPS) (Sigma-Aldrich) for 24 h prior to virus infection.

Positive control infected cells were prepared by infecting TC7 cells with SV40 strain 776-2E at 0.1 PFU/cell. After adsorption for 90 min, monolayers were washed with Tris buffered saline and then incubated in maintenance media (Eagles MEM, 2% FBS). Cultures were harvested at 72 h postinfection (p.i.) and DNA and RNA extracts were prepared as described [50–53].

### Real time quantitative PCR assays

Cells were processed for quantitative PCR (qPCR) analyses by a 60-min incubation at 55°C in proteinase K buffer (50 mM Tris-HCl, 2.5% Tween-20, Proteinase K 0.4 mg/ml), followed by a 10-min incubation at 95°C. qPCR assays to quantify SV40 DNA and human RNAseP copy numbers were performed as described [52,53] and viral genome copies per cell were calculated [52,54]. Assays to measure viral transcripts were described previously [53]. qPCR reactions were prepared in the PCR Clean Rooms Core Facility in the Department of Molecular Virology and Microbiology, Baylor College of Medicine [55]. Primer and probe sequences used for SV40 have been reported [52]. The RNAseP reagents were purchased (Applied Biosystems/Thermo Fisher Scientific, Foster City, CA).

### Limiting dilution-qPCR and ELDA analyses

Limiting dilution analyses were performed on DG75 and BJAB cells infected with SV40 strain 776-2E at an MOI of 5 PFU/cell. Cultures were passaged twice weekly. At times of harvesting, infected cells were washed and diluted in a background of uninfected cells of the same cell line into MicroAmp® optical 96-well reaction plates (Invitrogen/Thermo Fisher Scientific, Carlsbad, CA). Twelve replicates of each dilution were prepared, ranging from 200 to 0.1 virus-exposed cells per well. Cell pellets were processed and analyzed by qPCR as described above. Frequencies of SV40-positive cells were calculated after limiting dilution analysis at each harvest time point based on Poisson distribution statistics. Wells were scored as positive or negative for the detection of SV40 DNA and statistical calculations were completed using ELDA [56]. An ELDA calculator by Walter+Eliza Hall Institute for Medical Research Bioinformatics can be found at http://bioinf.wehi.edu.au/software/elda. Calculations for viral loads in positive cells were made using the SV40 genome copies detected per well in the qPCR assays.

### Virus rescue

Several virus rescue attempts and infectious center assays were performed to assess the possibility of recovery of infectious SV40 from SV40-infected lymphocytes. In one approach, 10^6^ infected cells were washed extensively and lysates were prepared by three cycles of freezing and thawing followed by low-speed centrifugation; dilutions of clarified lysates were inoculated in triplicate onto TC7 cell monolayers growing in 60-mm^2^ dishes. These cultures were harvested at various d.p.i. and analyzed by qPCR to measure viral DNA levels. Sometimes infected B cell lysates were assayed directly for plaque formation on TC7 cells to measure the level of infectious SV40. To examine viable cells using another approach, SV40 neutralizing antibody was added to some cultures following infection; 10^5^ viable infected cells were subsequently washed and plated onto permissive TC7 cell monolayers and incubated using minimal essential media (MEM) with 5% FBS [47]. These cocultured cells were harvested 5 and 10 days later and analyzed by qPCR for viral DNA. Finally, infectious center assays were performed using B cells infected with SV40 776-2E and 776-2E-SM1. These infected cells were plated directly onto TC7 monolayers and overlayed with agar media; plaques that formed were counted 15 days later [47].

### Flow cytometry

Intracellular expression of SV40 T-ag in live, infected cells was detected by flow cytometry using T-ag monoclonal antibody PAb416 (Santa Cruz Biotechnology, Santa Cruz, CA) as described previously [53]. For all samples, population densities in scatter plots determined to be cells were selected for gating to determine T-ag-positive cells. Median fluorescent intensity (MFI) values were calculated on these positive cells. To examine expression of cell surface markers on SV40-infected lymphocytes, live cells from infected cultures were treated with labeled, monoclonal antibodies [CD69–phycoerythrin (PE), CD80–isothiocyanate (FITC), CD86–allophycocyanin (APC), or CD25–PE] following the manufacturer’s recommendations (BD Biosciences Pharmingen™, San Jose, CA). A corresponding isotype control reaction was analyzed for each fluorochrome. Samples were analyzed in the Cytometry and Cell Sorting Core Facility at Baylor College of Medicine using a Beckman Coulter EPICS XL-MCL and FlowJo software (FlowJo, LLC, Ashland, OR).

### Statistical analysis

A general linear mixed model was used to compare SV40 DNA copies per cell and mean difference from mock between viral strains 776-2E and 776-2E-SM1 at each time point in a given cell line. The models included fixed effects for cell line, virus sample, and study time (discrete) as well as all two- and three-way interaction terms. The matrix of correlated residuals assumed a first-order autoregressive correlation structure. The model was used to estimate least squares means and to compare means between the two sample types. SV40 DNA copies per cell was log-transformed (natural logarithm) for analysis. To analyze cell proliferation between harvests, the mean cell increase for the mock group was computed for each time point. The difference from the mock mean was then computed for both viruses (776-2E and 776-2E-SM1). A general linear mixed model was also used to compare the change in SV40 DNA copies per cell over time. Fixed effects for this model were as described above except that study time was continuous and the model included a random intercept and slope for study time. Contrast statements were defined to test specific hypotheses about changes in SV40 DNA copies per cell over time. Statistical significance was assessed at the 0.05 level, and all analyses were completed using SAS version 9.4 (Cary, NC).

## Results

### Maintenance of SV40 genomes in cultures of human B cell lines

**Fig 1:**
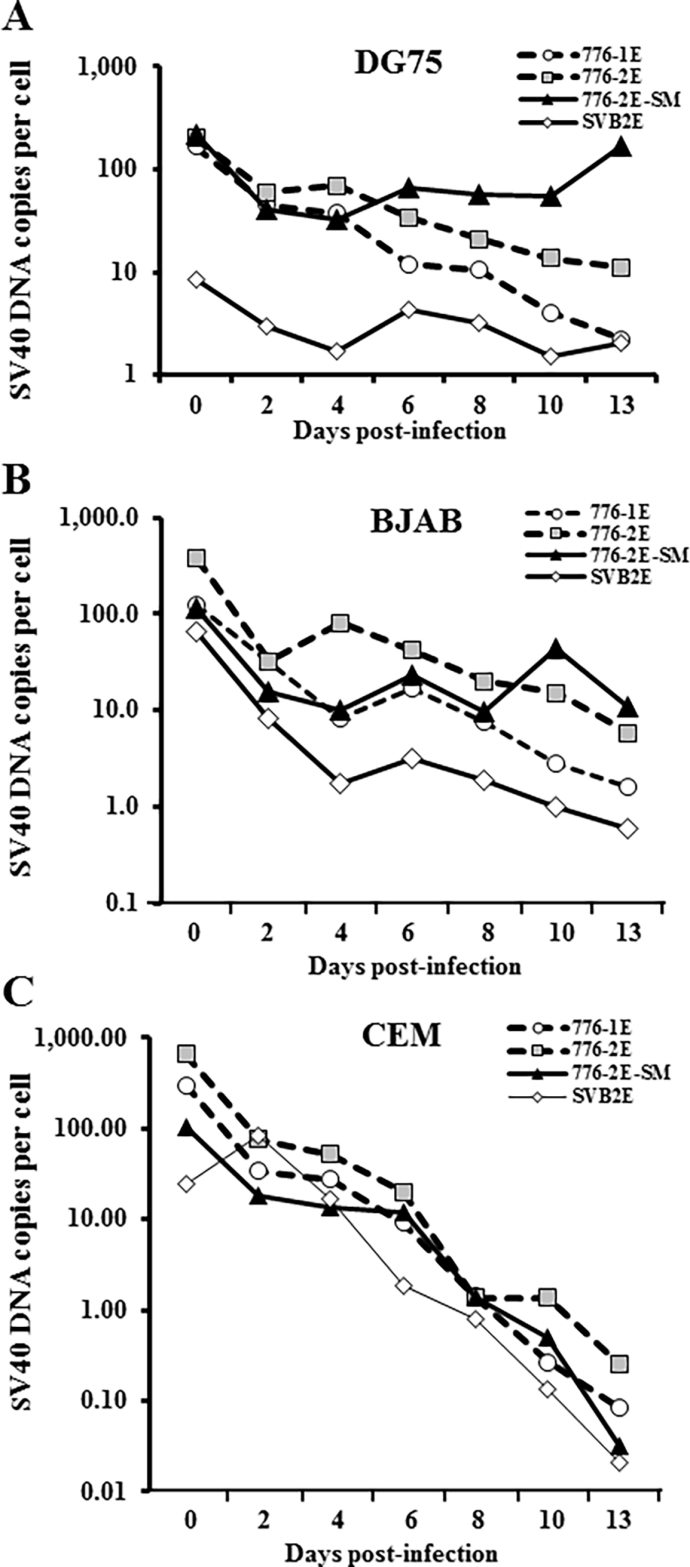
Maintenance of SV40 DNA in human lymphocyte cell lines. SV40 genomes per cell were quantified in human B cell lines (A) DG75 and (B) BJAB and in the T cell line (C) CEM following infections with SV40 strains 776-1E, 776-2E, SVB2E, and the viral miRNA-null mutant, 776-2E-SM1. Cells were harvested at the indicated d.p.i. and SV40 DNA was measured by qPCR. The human RNAse P gene, present at two copies per cell, was assayed to determine cell equivalents present. Values shown are SV40 DNA mean copy numbers per cell and are the average of four measurements for each target gene. These results indicate that SV40 genomes can persist at low levels in cultured human B cell lines.

Human lymphocyte cell lines were infected with SV40 to examine whether lymphocytes could support replication of the virus. Included in the initial investigations were two B cell lines, DG75 and BJAB, and the T cell line, CEM. SV40 viral strains tested included WT viruses 776-1E and 776-2E, with simple and complex RR, respectively, SVB2E (complex RR), and a viral mutant that fails to produce miRNA, 776-2E-SM1. Cells were infected as described in Materials and methods. During 13 days of observation, aliquots of cell cultures were collected at various d.p.i., were analyzed by qPCR for SV40 DNA and the human RNAseP gene, and the number of viral genome copies per cell calculated as described [52,54] (Fig 1). In the two B cell lines, viral genome levels decreased initially after infection and then stabilized between about 1–10 copies per cell throughout the 13-day time course (Fig 1A and 1B). Modestly higher levels of viral DNA were detected in cells infected with strain 776-2E (complex RR) compared to companion strain 776-1E with a simple RR. The highest levels of viral genomes at the end of the time course were detected in B cells infected by the miRNA mutant, 776-2E-SM1, with the DG75 cell line retaining higher levels of SV40 DNA than the BJAB cell line. In these B cell infections, the numbers of viral genomes retained were higher than that predicted by dilution calculations of input virus based on cell subcultures every 2 days (data not shown), suggesting limited viral DNA replication. In contrast, SV40 genome levels decreased continuously in the CEM T cell line (Fig 1C), at a rate of decrease predicted by the dilutions made during passage of the infected cells.

### Expression of SV40 large T-antigen (T-ag) in infected B cells

**Fig 2:**
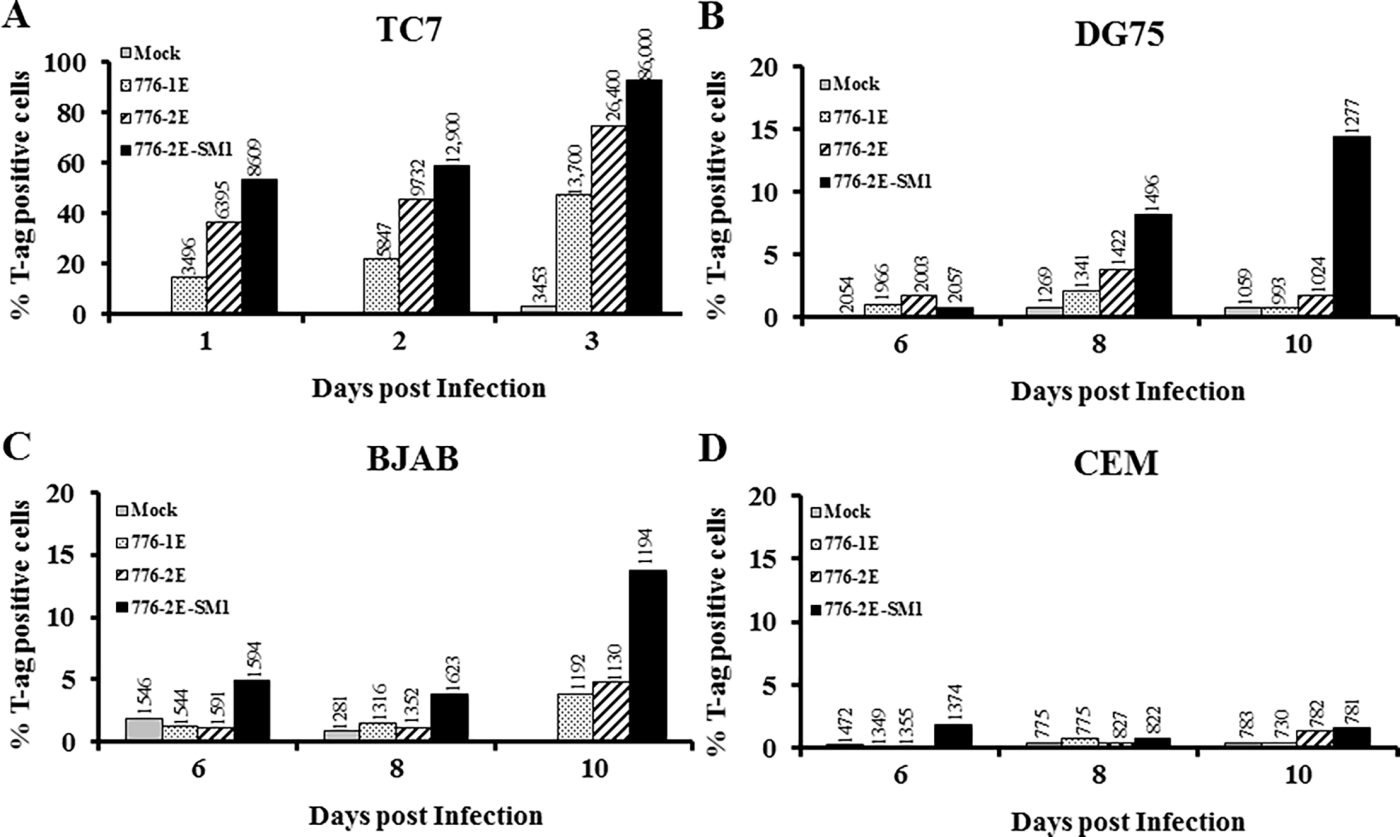
Expression of SV40 T-ag protein in human lymphocyte cell lines. Cells infected with SV40 strains 776-1E, 776-2E, or 776-2E-SM1 were analyzed by intracellular staining and flow cytometry for SV40 large T-ag. Anti-T-ag antibody PAb416 and goat anti-mouse F(abʹ)2-PE were used. The F(abʹ)2-PE reagent was used as a negative control on all samples (not shown). (A) TC7 cells, monkey kidney cells permissive for SV40 replication, (B) DG75 human B cells, (C) BJAB human B cells, (D) CEM human T cells. Percentages of cells expressing detectable SV40 large T-ag protein are shown. MFI values are indicated over each bar. These results show that detectable levels of SV40 large T-ag are expressed in some SV40-infected B lymphocytes.

Maintenance of SV40 genomes at low levels in human B cell lines provided the potential for viral gene expression. SV40 DNA replication is initiated by T-ag protein binding to the viral origin of replication. Therefore, flow cytometric assays were used to examine the possible expression of T-ag protein in SV40-infected lymphocytes as described in Materials and methods (Fig 2). Positive control samples for T-ag expression and detection were permissive TC7 monkey kidney cells infected with SV40 at 5 PFU/cell (Fig 2A). A higher percentage of TC7 cells expressed detectable T-ag following infection with 776-2E virus having a complex RR (75% positive cells at 3 d.p.i.), compared to strain 776-1E infection (43% positive cells at day 3). Even more T-ag-positive cells (97%) occurred in TC7 cells infected with the derivative miRNA mutant virus 776-2E-SM1 at day 3. For all three viruses, the percentage of T-ag-positive cells approximately doubled during the course of the experiment. The lower numbers of detectable T-ag-positive cells in the 776-1E-infected TC7 cell cultures likely reflected the limited sensitivity of the flow cytometric assay to measure low levels of nuclear T-ag in intact cells.

SV40 T-ag expression was detected in infected B-lymphocyte cell lines (6–10 d.p.i.), but at lower frequencies than in TC7 cells. Also, the percent positivity did not change much over time following viral infections, in contrast to observations in TC7 cells. The highest percentages of T-ag-positive cells (about 10–15%) were found in cells infected with mutant strain 776-2E-SM1 (Fig 2B and 2C). Whereas the DG75 and BJAB cell lines showed similar patterns of T-ag expression, infected CEM cells remained negative (Fig 2D). This latter observation is in agreement with the qPCR data, which indicated that viral genomes were poorly, if at all, retained in the CEM cultures.

MFI readings which indicate relative levels of T-ag increased over time in infected TC7 cells for all three viruses, rising more for 776-2E-SM1-infected cells than for the 776-2E and 776-1E infections. In contrast, MFI values indicated much lower levels of T-ag in infected B lymphocytes for all three viruses. In addition, MFI readings did not increase over time for the infected lymphocytes. These findings show that a low frequency of SV40-infected B cells express T-ag protein detectable by flow cytometry and at levels much lower than in permissive TC7 cells. These observations with infected TC7 cells demonstrate the influence of the structure of the SV40 enhancer (single vs. double) and of viral miRNA (presence vs. absence) on expression of T-ag in infected cells.

### Detection of SV40 in B cell populations following infection of primary human lymphocytes

**Fig 3:**
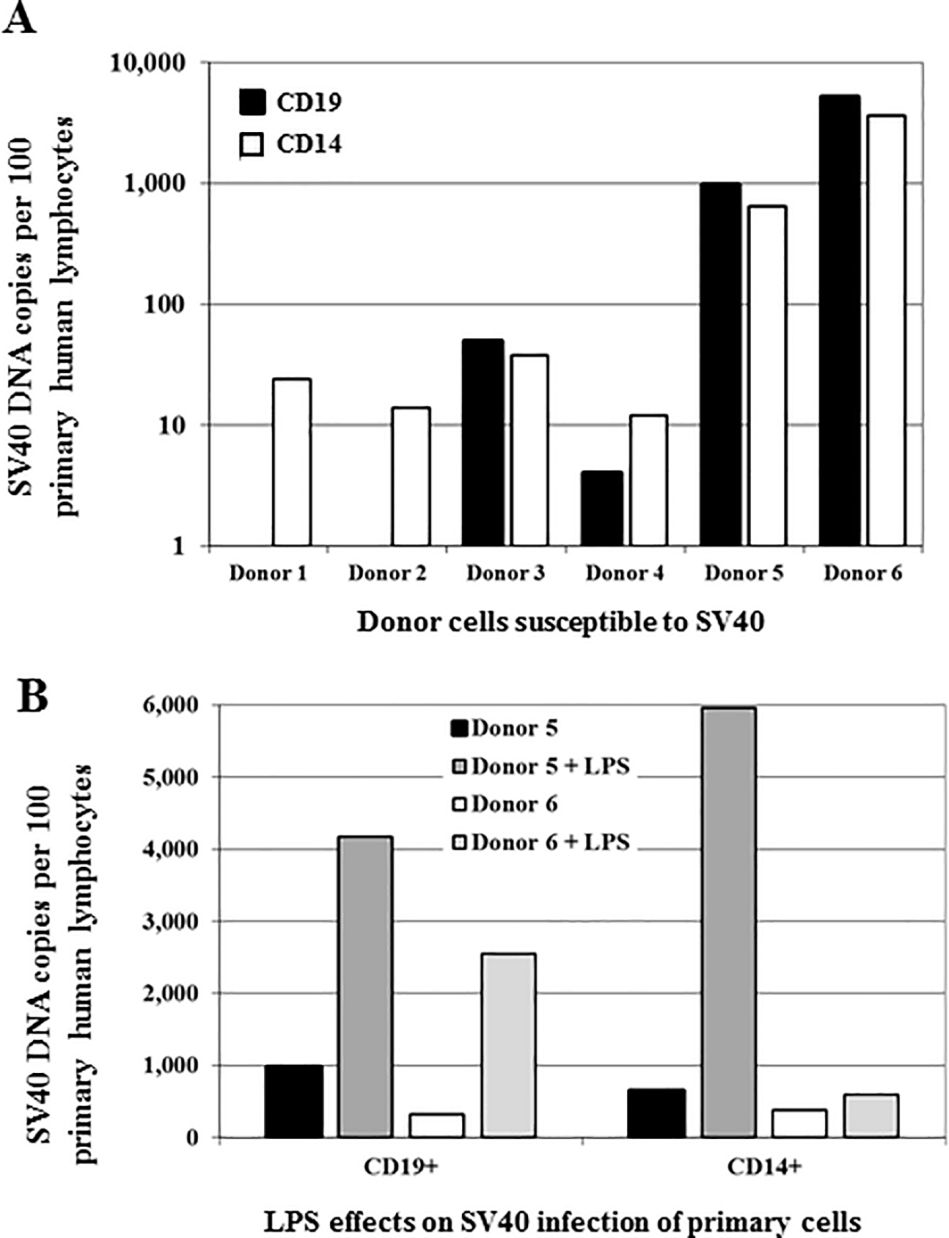
Infection of primary human lymphocytes by SV40. Primary human PBLs were obtained from normal donors (n = 6), were infected with WT SV40 strains, and cell types were separated by positive selection for surface markers CD19 (B cells), CD14 (monocytes/macrophages), and CD3 (T cells). SV40 DNA in selected lymphocyte subpopulations was measured by qPCR. Numbers of SV40 DNA copies detected per 100 cells assayed are shown. (A) Cells from donors 1, 2, 3 were infected with SV40 VA45-54-2E (MOI = 5) and analyzed at 7 d.p.i. Cells from donors 4 (VA45-54-2E), 5 and 6 (SVB2E) were infected (MOI = 1) and analyzed at 4 d.p.i. (donor 4) and at 6 d.p.i. (donors 5 and 6). SV40 DNA was not detected in CD19^+^ cells from donors 1 and 2 nor in CD3^+^ cells from donors 1–4; CD3^+^ populations were not isolated from donors 5 and 6 (data not shown). Studies of different donor cells were individual experiments. (B) PBLs from donors 5 and 6 were either untreated or activated with LPS (1 μg/ml) for 24 h, then infected with SV40 (SVB2E, MOI = 1). Cell populations were separated at 6 d.p.i. (donor 5) and 8 d.p.i. (donor 6) and were analyzed by qPCR for SV40 DNA. These results show that CD19^+^ and CD14^+^ subsets of primary human lymphocytes can be infected by SV40.

Retention of SV40 DNA and low level expression of T-ag in established lymphocyte cell lines suggested that B cells could support initial steps of SV40 infection. To ascertain whether those observations were applicable to primary cells or were unique to the specific lymphocyte cell lines used, primary human lymphocytes were examined. PBLs were isolated from the blood of a total of six normal donors and were infected with SV40. At 4–7 d.p.i., the B, T, and monocyte cell populations were isolated by positive selection using magnetic beads for CD19^+^ cells, CD3^+^ cells, and CD14^+^ cells, respectively, and were assayed for SV40 DNA (Fig 3). SV40 DNA was detected in both the CD19^+^ B cell and CD14^+^ monocyte/macrophage subpopulations from four donors, whereas SV40 was not found in the CD19^+^ B cells from 2 donors (Fig 3A) nor in any of the CD3^+^ T cells following infection (data not shown). As SV40 DNA was not detected in B cells from two of the six donors following viral exposure, this suggests there may be variation in susceptibility to virus infection among individuals. These results with primary human lymphocytes are consistent with those described above in which SV40 infection resulted in measurable viral DNA in human B lymphocyte cell lines.

Unlike established cell lines, primary lymphocytes are not actively dividing in culture, so the effect of activation stimulation of the primary cells on viral genome levels was examined. PBLs from two donors were treated with LPS and infected with SV40 (see Materials and methods). Six to eight days later cells were separated into subpopulations and qPCR analyses were performed. Increased numbers of viral genomes were detected in cultures of activated primary B cells (CD19^+^) from both donors and in activated monocytes (CD14^+^) from one donor compared to parallel nonactivated cultures (Fig 3B). In contrast, SV40 DNA remained undetectable in CD3^+^ T cells. These results suggest that the maintenance and/or replication of SV40 genomes carried by human B cells may be sensitive to the state of those cells.

### Frequency of maintenance and levels of SV40 genomes retained in B cell lines

Detection of SV40 DNA following viral infection indicated that SV40 genomes persisted in cultures of infected human B cell lines at ~1–10 viral genomes per cell (Fig 1). Such whole-culture derived data do not differentiate between the possibilities that either many B lymphocytes in a culture contain a low number of viral genomes or that a few cells carry or replicate viral DNA at higher levels. To determine more accurately the frequency of cells containing SV40 DNA and the viral copy numbers in those infected B lymphocytes, limiting dilution qPCR (LD-qPCR) and Extreme Limiting Dilution Analysis (ELDA) were performed. This technique allows quantitation of the frequency of cells that have a particular characteristic. DG75 and BJAB cells were infected with SV40 strain 776-2E, were harvested at various times p.i., and were analyzed by qPCR following limiting dilution as described in Materials and methods. Reactions were scored as positive or negative and frequencies of SV40-positive cells were calculated based on Poisson distribution statistics (Table 1). For DG75 cells at 10 d.p.i., approximately 1 in 3.3 cells (95% confidence interval 2.1 to 5.6), or ~30% of cells, contained SV40 DNA. One week later at 16 d.p.i., the frequency was 1 in 3.0 cells (95% confidence interval 1.8 to 4.7), or ~33% positive cells. To determine the viral loads in cells containing SV40 genomes, calculations were made using the SV40 genome copies detected per well in the qPCR assays. For DG75 cells, the average of the viral loads measured at 10 and 16 d.p.i. indicated that 1.6 (or from 1 to 2) viral genomes were present per infected cell.

**Table 1:** Frequency of maintenance and level of viral loads in SV40-infected human B cell lines DG75 and BJAB.

Cell line	dpi	No. cells from SV40-infected cultures plated^a^	No. replicates positive^b^	Estimated frequency of positive cells^c^	No. SV40-positive cells per well^d^	Average SV40 genomes per well^e^	SV40 genomes per positive cell^f^
DG75	3	50	12	1 in 1.9	27	772	29
		10	12	(3.0–1.9)	6	83	13
		5	10		3	10	3
		1	10				
		0.5	0				
	10	50	12	1 in 3.3	16	31	1.9
		10	12	(5.6–2.1)	3	5	1.7
		5	9		1	2	2
		1	3				
		0.5	1				
	16	50	12	1 in 3.0	16	10	0.6
		10	12	(4.7–1.8)	3	4	1.3
		5	12		1	2	2
		1	0				
		0.5	0				
					[Avg SV40 genomes/DG75 cell = 1.6^g^]		
BJAB	3	50	12	1 in 2.7	18	80	4
		10	12	(4.4–1.7)	3	16	4
		5	9		1	7	4
		1	3				
		0.5	1				
	6	50	12	1 in 24.1	2	6	3
		10	2	(40.3–14.4)	0.4	0.5	1
		5	2		0.2	0.3	2
		1	0				
		0.5	0				
	10	50	2	1 in 29.2	2	0.5	0.3
		10	1	(49.5–17.2)	0.3	0.5	1
		5	1		0.2	0.3	2
		1	0				
		0.5	0		[Avg SV40 genomes/BJAB cell = 1. 6^g^]		

^a^Number of cells from SV40-infected cultures plated in each well of one row. Strain 776-2E was used for infections (5 PFU/cell).

^b^Number of wells out of 12 that were positive for SV40 DNA by qPCR.

^c^Estimated frequency of SV40-positive cells calculated using the ELDA calculator, reported as the number of uninfected cells for each infected cell.

^d^Number of SV40-positive cells in each well calculated by *a/c*.

^e^Average SV40 genomes/well detected by qPCR in the positive wells of 12 replicates.

^f^Number of SV40 genomes in a positive cell calculated by *e/d*.

^g^Average SV40 genomes/positive cell were calculated for DG75 cells using viral load data from 10 and 16 d.p.i. Calculations for BJAB cells used viral load data from 6 and 10 d.p.i. Calculations for both cell systems averaged 1.6 viral genomes/positive cell.

Relatively fewer infected BJAB cells retained SV40 DNA during culture (Table 1). At 3 d.p.i, 1 in 2.7 cells were estimated to be virus-positive (95% confidence interval 1.7 to 4.4), or approximately 37%. This value dropped to 1 in 24.1 cells (95% confidence interval 14.4 to 40.3) (4% positive cells) at 6 d.p.i. and 1 in 29.2 cells (95% confidence interval 17.2 to 49.5) (3% positive cells) at 10 d.p.i. Although the frequency of BJAB cells carrying SV40 DNA was lower than with the DG75 cells, the average viral loads at 6 and 10 d.p.i. were calculated to be similar with 1.6 (or 1–2) viral DNA copies per infected cell. These values are compatible with the concept that persistently infected human lymphocytes carry small numbers of SV40 viral genomes per cell.

### Effects of SV40 infection on human B cell proliferation and viability

**Fig 4:**
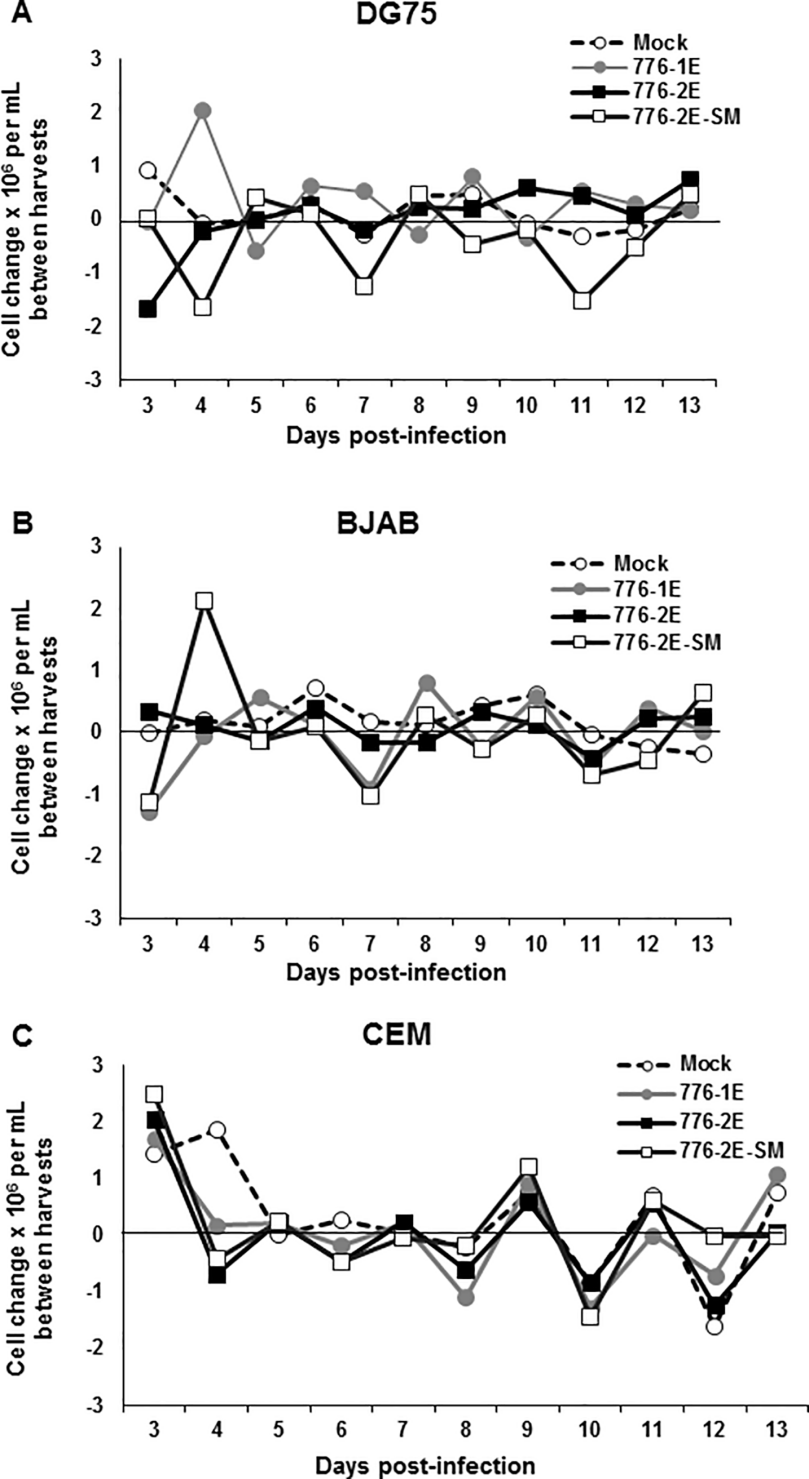
Effects of SV40 infection on proliferation of human lymphocyte cell lines. Human B cells (A) DG75 and (B) BJAB and T cells (C) CEM were infected with SV40 strains 776-1E, 776-2E, and the miRNA mutant 776-2E-SM1. Cells were collected at indicated time points after infection and live cells were determined by trypan blue exclusion. After counting, cell densities were adjusted to 1 × 10^6^ cells/ml for the next incubation period. Changes in cell numbers between harvests are plotted. Both DG75 and BJAB cells infected by the 776-2E-SM1 miRNA-null mutant showed somewhat slowed growth compared to the mock-infected cells, but remained viable.

To investigate if the presence of the virus, the viral miRNA, or viral RR differences might influence cell growth, the proliferation of cells during an infection time course was measured. Changes in total live cell numbers following infection of three lymphocyte lines with three variants of SV40 were compared to the growth of matched mock-infected control cultures between harvests. At each harvest, total live cells were counted and then the cell density was reset to 1 × 10^6^ cells/ml for the next incubation period. Changes in cell numbers per ml of media between harvest times for the cultures were calculated (Fig 4). Infection of DG75 and BJAB cells with WT strains 776-1E (simple enhancer) and 776-2E (complex enhancer) had little effect on proliferation of the cells over the 2-week experimental period relative to the growth of mock-infected cultures. In contrast, the growth of both B cell lines was somewhat reduced following infection by the miRNA mutant 776-2E-SM1 (Fig 4A and 4B). Proliferation of the CEM T cells was not affected (Fig 4C). The reduction in total numbers of live cells of mutant SM1-infected B cells (compared to mock-infected cells) was not due to cell death as the percentage of viable cells for all SV40-infected and mock-infected lymphocyte lines remained above 85% throughout the course of the experiment (data not shown). These observations suggest that SV40 infections of human B cells are generally not cytolytic.

### Rescue of infectious SV40 from persistently infected human B cell lines

**Fig 5:**
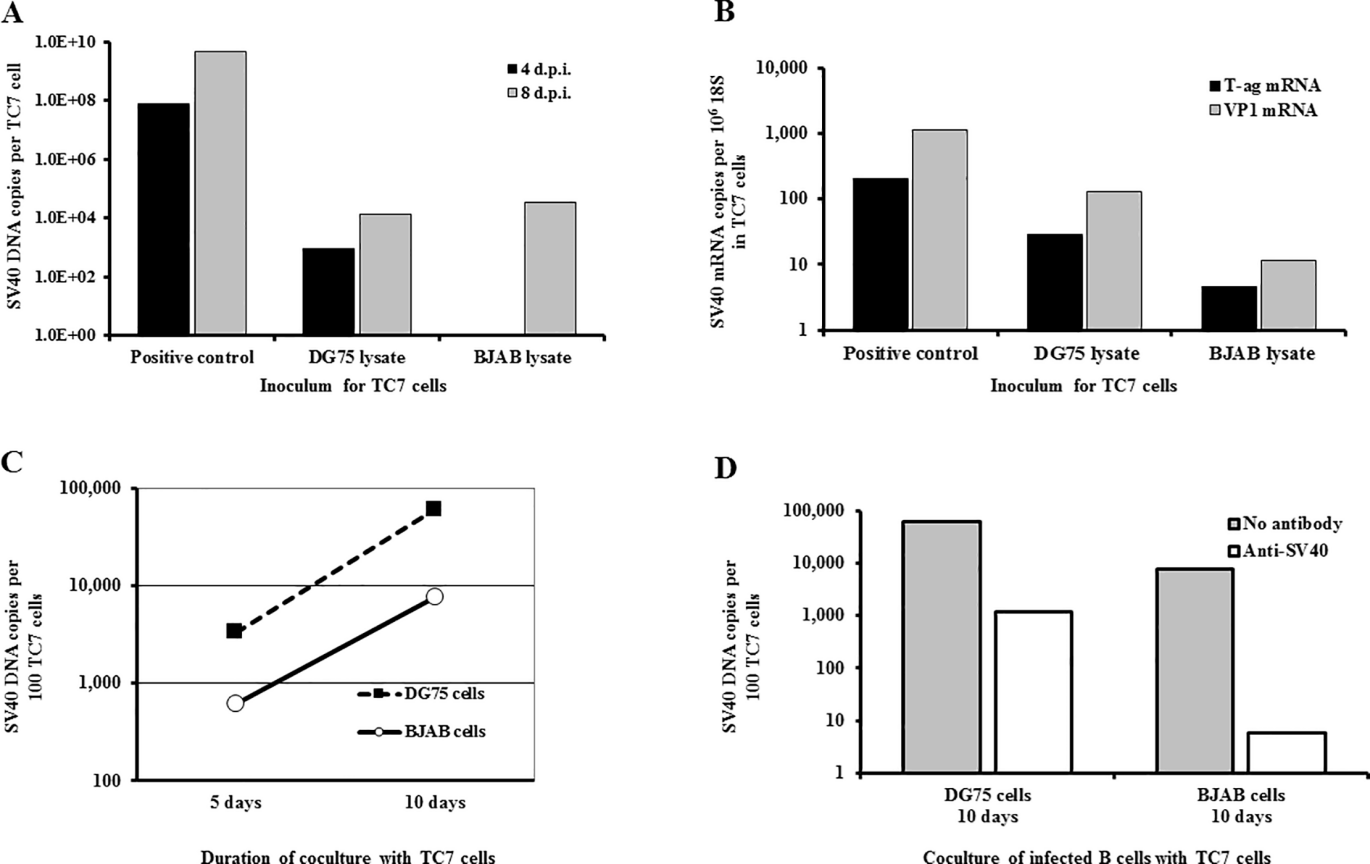
Rescue of infectious SV40 from SV40-infected human B cells. DG75 and BJAB cell lines were infected with SV40 strain 776-2E at 5 PFU/cell and washed. (A) Lysates of infected B cells were prepared 10 days later and the equivalent of 10^6^ cells was inoculated onto monolayers of permissive TC7 cells. The TC7 cells were harvested at 4 and 8 days following exposure to the B cell lysates and were analyzed by qPCR for SV40 DNA. Positive control samples were lysates of TC7 cells infected with SV40 776-2E. SV40 DNA was detected in cultures of TC7 cells exposed to lysates of infected DG75 and BJAB cells. (B) TC7 cells harvested at 8 days after exposure to the cellular lysates were analyzed by reverse transcriptase PCR for expression of SV40 transcripts. Both early (T-ag) and late (VP1) SV40 mRNAs were detected. (C) At 6 d.p.i. viable DG75 and BJAB cells were collected and washed, and 10^5^ cells were plated onto TC7 monkey kidney cell monolayers. At 5 and 10 days after coculture, samples of cells were extracted and assayed by qPCR. SV40 DNA was detected in the cocultures of TC7 and live DG75 or BJAB infected cells. The increase in viral DNA levels between 5 and 10 days of coculture indicated active viral replication. (D) Parallel cultures of SV40-infected DG75 and BJAB cells were treated with SV40 neutralizing antibody following infection or were left untreated. At 6 days after infection live cells were cocultured with TC7 cells in the absence of antibody. The cocultures were analyzed 10 days later for SV40 DNA. These approaches showed that both lysates of SV40-infected human B cells and live infected human B cells can transmit infectivity competent for viral DNA replication and viral transcription to susceptible cells.

The qPCR and LD-qPCR analyses indicated that SV40 genomes persisted in B cells at low levels; flow cytometry showed that SV40 T-ag protein was expressed at detectable levels in a low percentage of those cells; and cell growth and viability data indicated that SV40 was not causing lytic infections in the B cells. We next investigated whether the viral DNA persisting in the B cells could generate infectious virus. Three different virus rescue approaches were undertaken. First, lysates of infected B cells were tested for infectivity in susceptible monkey kidney TC7 cells (Fig 5). DG75 and BJAB cells were infected with SV40 776-2E at 5 PFU/cell and stringently washed three times as described in Materials and methods. Ten d.p.i. the cells were harvested and subjected to three cycles of freezing and thawing. The clarified lysates from 10^6^ cells were inoculated onto TC7 cell monolayers which were harvested at two later time points and analyzed by qPCR for SV40 DNA levels. SV40 DNA was detected in the recipient TC7 cell cultures that increased between 4 and 8 days following the addition of infected cell lysates (Fig 5A). These results showed that viral infectivity was transferable from the infected B cells to permissive TC7 cells, where complete SV40 replication occurred. The amount of infectivity transferred appeared to be small as the level of viral DNA induced by B cell lysates at 8 d.p.i. was several logs lower than that induced by a positive control lysate from SV40-infected TC7 cells. Both early (T-ag) and late (VP1) viral transcripts were detected in the TC7 cells 8 days after exposure to lysates of infected B cells (Fig 5B). Levels of late (VP1) mRNA induced by the lysates were more abundant than that of early (T-ag) transcripts. This difference in relative abundance is normally observed during SV40 replication, as seen with the positive control lysate. These results substantiate the ability to recover infectious SV40 from infected B cells.

The second attempt to rescue infectious virus involved coculture of infected B cells with permissive TC7 cells. Six days after infection with SV40 strain 776-2E 10^5^ live DG75 or BJAB cells were washed and then plated onto TC7 cell monolayers and incubated under media. After 5 and 10 days of coculture, the cultures were harvested and extracts were analyzed by qPCR for SV40 DNA (Fig 5C). Viable cells of both B cell lines were capable of transmitting viral infectivity to TC7 cells; active replication of SV40 was evidenced by an increase in viral DNA copies in the cocultures between 5 and 10 d.p.i. To minimize possible residual infectious virus from the original inoculum that might remain associated with the exterior of infected B cells despite extensive washes, parallel lymphocyte cultures were exposed to SV40 neutralizing antibody throughout the initial infection period prior to coculturing. Antiserum was not added when the viable B cells were plated onto TC7 monolayers. Infectious virus was recovered from the antibody-treated B cells, but at somewhat reduced levels compared to the untreated cultures (Fig 5D). These data indicate that the virus rescues were not due simply to the retention of residual inoculum, although they do not rule out the theoretical possibility that the rescued virus represented original input virus that had been internalized by the B cells but never uncoated. Considering the reduced level of virus from the treated cultures, it is possible that the antibody neutralized some newly replicated virus in the original infected B cell cultures, reducing the number of virus-infected cells added to the TC7 cells and the subsequent virus yield. Also, it cannot be ruled out that carry-over of neutralizing antibody from the original B cell cultures into the cocultures had some inhibitory effects on replication of rescued virus in the TC7 cells.

The third approach employed infectious center assays to estimate the fraction of live infected B cells able to initiate viral replication in permissive cells. DG75 and BJAB cells were infected with SV40 and 9 days later live cells were collected and washed and then plated onto TC7 cell monolayers and the monolayers overlayed with an agar mixture. Plaque formation would develop following infectious virus transfer from live infected lymphocytes to the TC7 cells. SV40 plaques were counted 15 days later (Table 2). Generally less than 100 cells per million infected DG75 or BJAB cells (<0.01%) formed infectious centers under the conditions of the assay. Whereas the LD-qPCR analyses estimated SV40 persistent infection frequencies of about 30% and 3%, respectively, in DG75 and BJAB cells (Table 1), it appears that it is the rare infected B cell that initiates complete viral replication detectable in infectious center assays.

**Table 2:** Infectious center formation by live SV40-infected human B cells^a^.

B cell line	Virus infection	No. infectious centers (PFU/10^6^ cells)^b^
DG75	776-2E	52
	776-2E-SM1	36
BJAB	776-2E	103
	776-2E-SM1	44

^a^Live infected B cells at 9 d.p.i. were plated onto TC7 cells and an agar overlay added for plaque assay.

^b^Plaques that formed were counted 15 days post-plating and the number of PFU (infectious centers) per 10^6^ B cells calculated. Numbers are averages of 7–9 assay plates.

### Long-term persistence of SV40 DNA in human B lymphocytes

Experiments were next undertaken to address the characteristics of longer-term SV40 persistence in lymphocytes. To explore possible ranges in patterns of persistence, two additional human cell lines were tested (TF-1a and RL) in addition to DG75. For these studies, viral infections by the WT virus, 776-2E, and the miRNA-null mutant, 776-2E-SM1, were compared. Initial infections were at 5 PFU/cell. Cells were subcultured every 3–5 days. Samples were removed at different time points during 28-day experiments, were analyzed by qPCR, and the number of viral genome copies per cell calculated.

**Fig 6:**
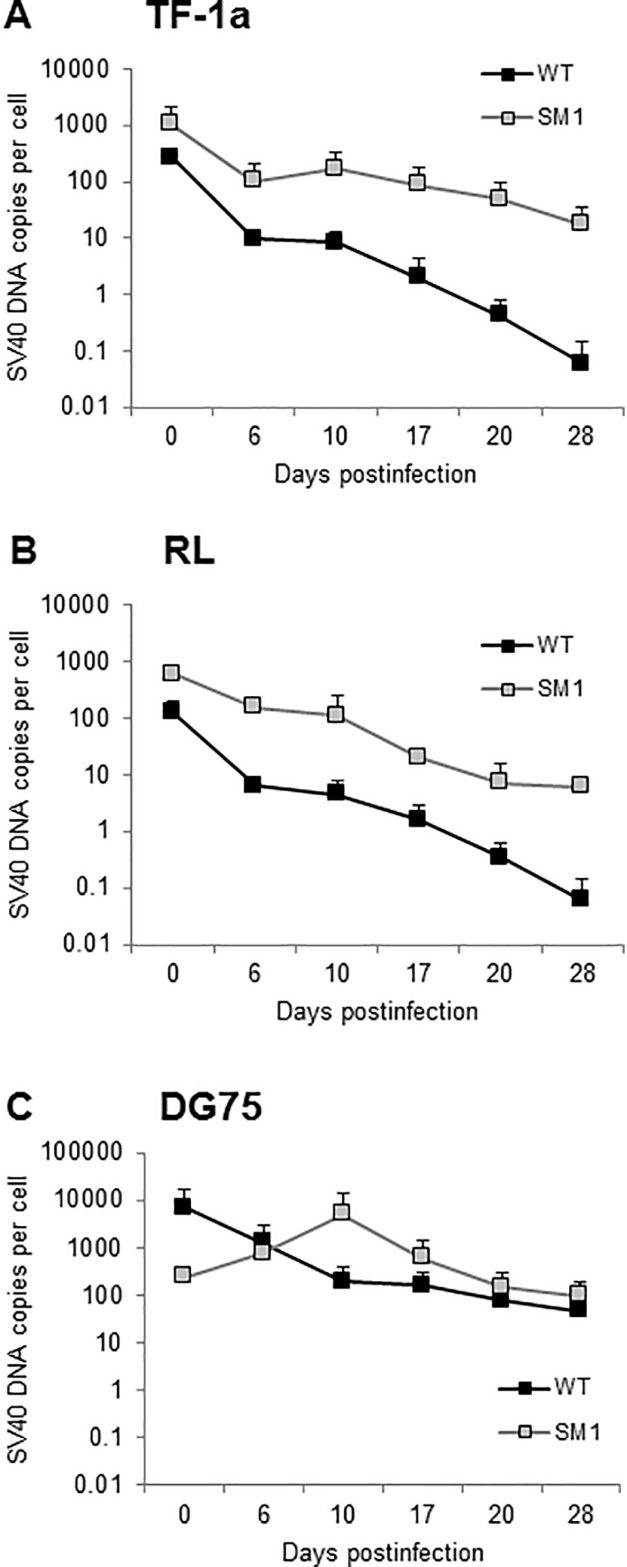
Long-term retention of SV40 viral DNA in human myeloid cells and B cells following viral infection. Human myeloid cells [(A) TF-1A] and B cells [(B) RL and (C) DG75] were infected with SV40 776-2E or with the 776-2E-SM1 miRNA mutant virus at 5 PFU/cell. Samples were harvested at the indicated d.p.i. and viral DNA and the human RNAse P gene (present at two copies per cell) were measured by RQ-PCR. Graphs show SV40 DNA mean copy numbers/cell. Each value represents the average SV40 viral load from at least two independent experiments with two measurements for each target gene; error bars show + and–one standard error. These results show that SV40 DNA can persist in human B cells and myeloid cells over long periods of time.

Similar patterns of viral persistence were found with the TF-1a (myeloid) and RL (B lymphocyte) cell lines (Fig 6A and 6B). WT virus (776-2E) DNA copies decreased over time and by 28 d.p.i. were still detectable, but at less than one copy per cell. In contrast, following infection with the 776-2E-SM1 mutant, viral loads remained at about 10 SV40 DNA copies per cell in both cell lines at 28 d.p.i. In the TF-1a cells, this difference between the levels of WT and SM1 viral DNA was significant (*p* < 0.05) at 17 d.p.i. and thereafter. In the RL cells, the retention of 776-2E-SM1 DNA was significantly higher than that of the WT virus from days 6 through 28 (*p* < 0.05). In contrast, in the DG75 cells, there were no significant differences between viral loads for the two viruses throughout the 28-day experiment (Fig 6C). It was noted that viral loads for 776-2E were greater in the DG75 cells than detected in the other cell lines tested and remained at about 50 DNA copies per cell at the termination of the experiment. The 776-2E-SM1 mutant-infected DG75 cultures contained higher levels of viral DNA, averaging about 100 copies per cell at day 28. A similar pattern between the WT and mutant viruses was observed in the short-term study in DG75 cells (Fig 1A). A relative rise in SM1 DNA copies over that of WT virus was seen at 10 and 13 d.p.i. in both the shorter study and longer study, which then gradually decreased in the long-term study (Fig 6C). These fluctuations probably reflect slight differences in initial infections or in cell culture conditions.

Biostatistical analysis using a general linear mixed model of changes in SV40 DNA copies per cell over time found that significant changes in viral retention were dependent on both the virus type (*p* = 0.0002) and the cell line (*p* = 0.0005). Analyses found no difference in viral retention between the RL and TF-1a cells for either WT (*p* = 0.32) or SM1 (*p* = 0.61) viruses. However, when comparing SV40 infections in RL and DG75 cells, the level of viral retention was significantly different both for WT (*p* = 0.004) and SM1 (*p* = 0.017) viruses. Similarly, when TF-1a and DG75 cells were compared, there were significant differences in viral retention for both WT (*p* = 0.0006) and SM1 (*p* = 0.05) viruses. These results show that human lymphoid cell lines can differ in their responses to SV40 infection. These data confirm observations with primary PBLs (Fig 3) that CD19^+^ B lymphocytes can be infected with SV40 and show that SV40 can persist at variable levels in human B cells and in some myeloid cells over relatively long periods of time. SV40 miRNA (encoded by WT virus 776-2E) appears to reduce viral DNA loads persisting in those cells.

### Long-term effects of SV40 infection on human myeloid and B cell proliferation and viability

**Fig 7:**
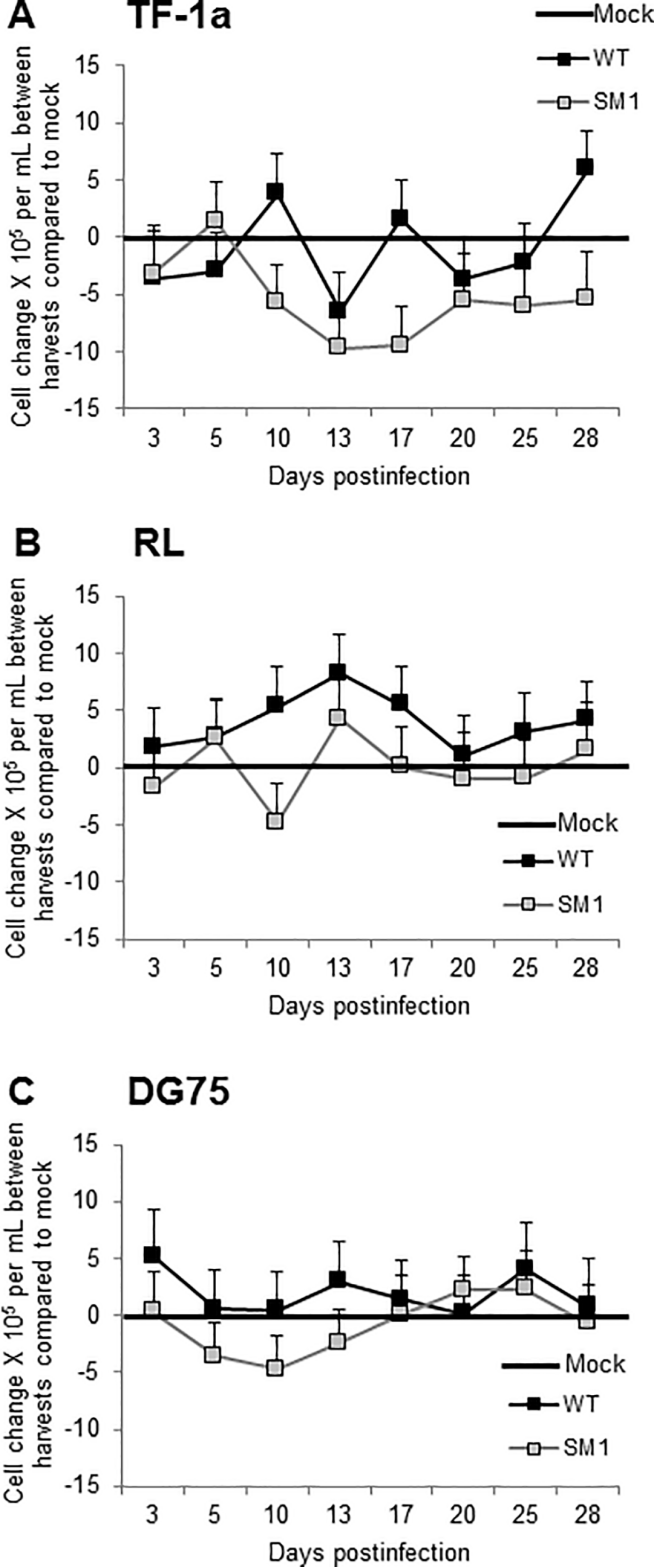
Human myeloid and B cell proliferation following viral infection. Human cells (A) TF-1a, (B) RL, and (C) DG75 were infected with SV40 776-2E and with the 776-2E-SM1 miRNA mutant virus at 5 PFU/cell. Samples were harvested at the indicated d.p.i. and live cells were quantified using a trypan blue exclusion assay. After counting, the cell density was then reset to 1 × 10^6^ cells/ml for the next incubation period. Changes in cell numbers between harvests are plotted relative to the growth of mock-infected cells (set at zero). Each data point is the average of at least two independent experiments; error bars show + and–one standard error. These results indicate that human B cells and myeloid cells can continue to proliferate following SV40 infection.

Following infections with SV40 WT and mutant SM1 viruses, the human cells were monitored at each time point for growth and viability using a trypan blue exclusion assay. As described above (Fig 4), total live cells were counted and the cell density adjusted at each harvest time point. Changes in cell numbers per ml of media between harvest times for virus-infected cultures were plotted relative to the growth of mock-infected cells (Fig 7). All three cell lines [myeloid TF-1a (Fig 7A), B cell RL (Fig 7B), B cell DG75 (Fig 7C)] continued to proliferate following SV40 infection. Although occasional harvests showed a statistically significant difference (*p* < 0.05) between the growth of the two virus-infected cell cultures of a given cell line (TF-1A: days 10, 17, 28; RL: day 10), the overall trend was that the two virus-infected cultures proliferated similarly (*p* > 0.05). It was noted that virus-infected TF-1a cells generally grew less well than the mock-infected culture and that the mutant-infected TF-1a cells tended to proliferate more slowly than the WT-infected cultures. It appeared that in the three human cell lines tested, SV40 exposure did not lead to significant cell destruction or loss of growth potential. This same outcome was observed in shorter-term proliferation experiments (Fig 4).

**Fig 8:**
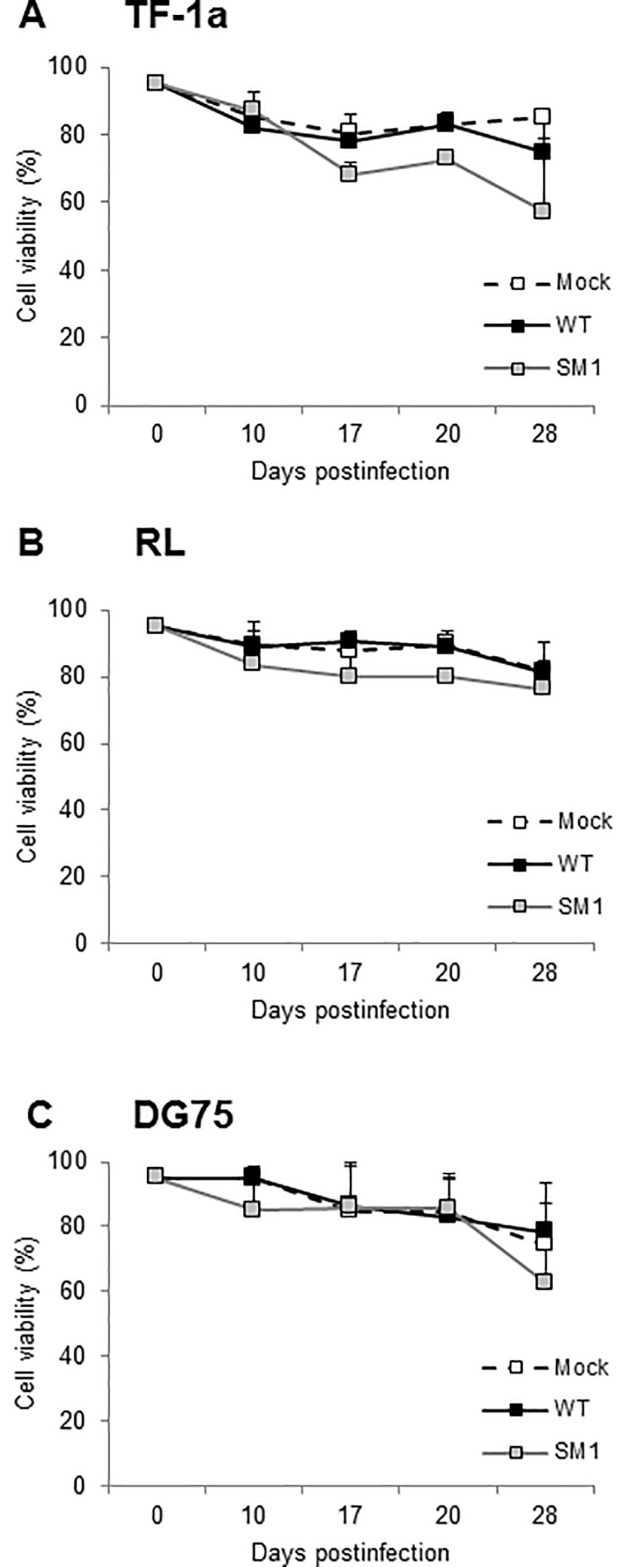
Cell viability during long-term SV40 infection of human myeloid and B cells. Human myeloid cells [(A) TF-1a] and B lymphocytes [(B) RL and (C) DG75] were infected with SV40 776-2E and with the 776-2E-SM1 miRNA mutant virus at 5 PFU/cell. Samples were harvested at the indicated d.p.i. and both total and viable cell numbers were determined in a trypan blue exclusion assay. Each data point is the average of at least two independent experiments; error bars show standard deviation. These results show that the majority of SV40-infected human lymphoid cells remain viable.

The human cell cultures were also measured for cell viability in a trypan blue exclusion assay. The majority of cells in the virus-infected cultures remained viable through 28 d.p.i. (Fig 8), confirming that SV40 infections of human B cells and myeloid cells were predominantly noncytolytic in nature. The somewhat lower cell viability associated with the 776-2E-SM1 mutant virus infection observed at some time points, compared to SV40 WT infection, might be an indication that increased expression of the T-ag protein could reach damaging levels in some human B cells and myeloid cells or that the cell death could be attributable to unknown effects of SV40 infection on those cells in the absence of the viral miRNA.

Based on these month-long observations, SV40 DNA persisted in infected human B lymphocytes over multiple cell generations and the cells remained viable. The results support the conclusion that SV40 viral infection of human B cells is predominantly persistent, nonproductive, and noncytolytic in nature.

### Recovery of infectious SV40 from long-term infected human B cells

Infected human myeloid (TF-1a) and B cell lines (RL, DG75) were evaluated periodically to determine if infectious SV40 could be detected. Cell lysates were prepared and tested directly for plaque-forming ability on TC7 cells (Table 3). Infectious virus was recovered from all infected cultures through the first 10–12 d.p.i. with levels of WT 776-2E virus infectivity being lower than that of the miRNA-null mutant. As time progressed, levels of WT infectious virus fell below the limits of detection of the assay (<1 × 10^1^ PFU/10^6^ cells). In contrast, miRNA mutant infectivity remained at low but detectable levels through day 28. The explanation for this difference is unknown. It is possible there is higher production of infectious virus in some cells due to increased levels of T-ag protein present in mutant-infected cells. It is also possible that some unknown effect of the viral miRNA on a cellular process modulates virus replication and the absence of the miRNA results in higher viral replication. These findings are consistent with analyses at shorter times p.i. (Fig 5, Table 2) that indicated that low levels of infectious SV40 could be recovered from some infected B cells. They confirm the interpretation that SV40 infections in human B cells are predominantly nonproductive but can occasionally yield infectious virus.

**Table 3:** Detection of infectious SV40 in cell lysates of virus-infected B cells and myeloid cells by plaque assay^a^.

Human cell line	Virus^b^	Days postinfection of human cells	Cell lysate PFU/10^6^ cells
TF-1a	776-2E	12	1.7 × 10^2^
		20	<1 × 10^1^
		28	<1 × 10^1^
	776-2E-SM1	12	1.3 × 10^3^
		20	9.8 × 10^2^
		28	9.8 × 10^1^
RL	776-2E	10	2.9 × 10^3^
		20	<1 × 10^1^
		28	<1 × 10^1^
	776-2E-SM1	10	7.5 × 10^4^
		20	1.6 × 10^3^
		28	1.5 × 10^1^
DG75	776-2E	10	2.0 × 10^1^
		20	<1 × 10^1^
		28	<1 × 10^1^
	776-2E-SM1	10	3.4 × 10^5^
		20	6.7 × 10^4^
		28	6.0 × 10^3^

^a^Cell lysates of 10^6^ infected human cells were prepared at various d.p.i. by freezing and thawing and the lysates tested for plaque-forming ability on TC7 cells. Plaques were counted 15 days later and the number of PFU (infectious virus) per 10^6^ B cells/myeloid cells calculated. Data are from two to three independent experiments.

^b^Mock-infected cultures harvested at the same time points were all virus-negative (<1 × 10^1^ PFU/10^6^ cells).

### Effect of SV40 infection on expression of lymphocyte surface markers

**Fig 9:**
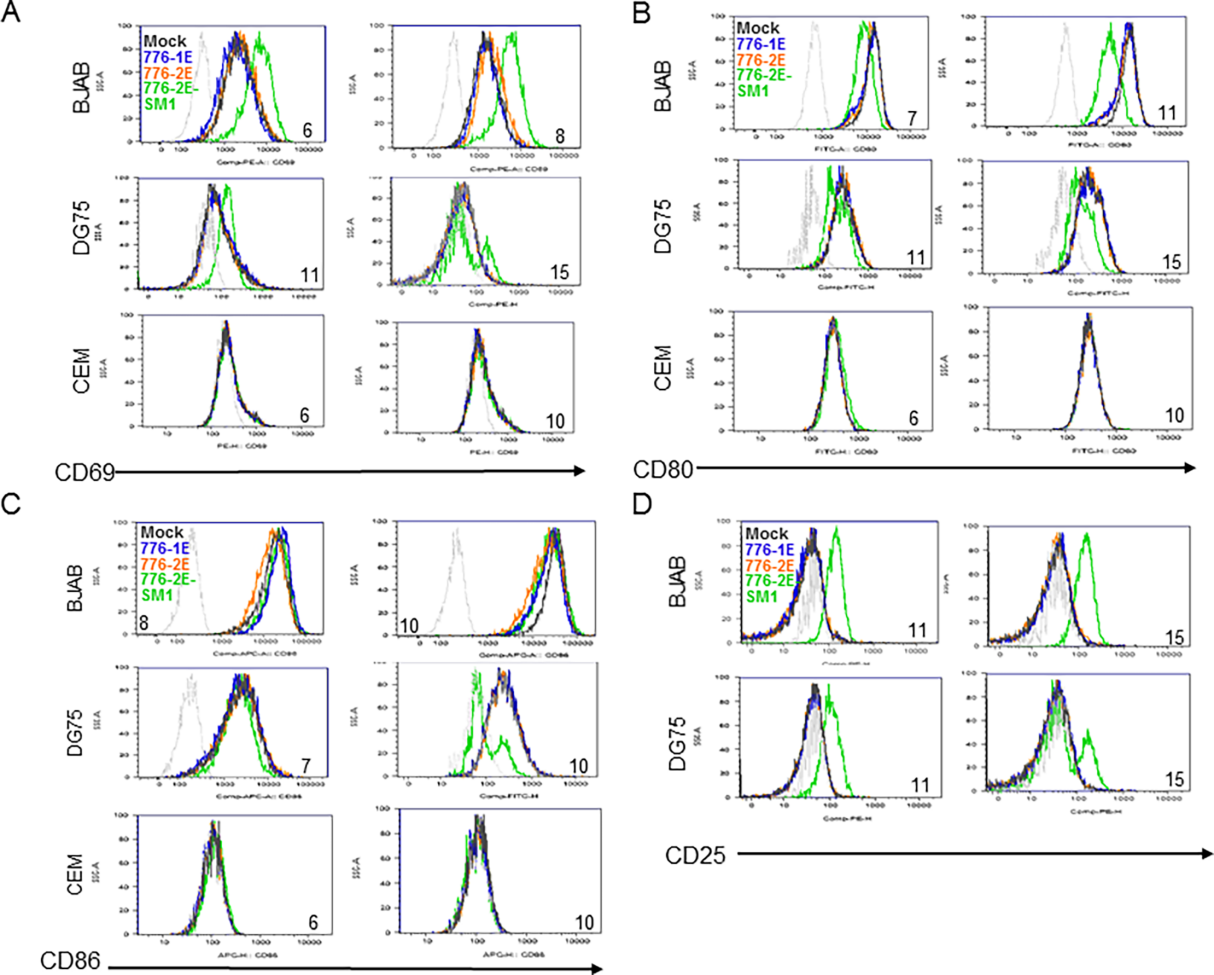
Effects of SV40 infection on expression of surface markers on human B cells. Surface expression levels of lymphocyte activation markers (A) CD69 and (D) CD25, and of costimulation molecules (B) CD80 and (C) CD86, were examined by flow cytometry. BJAB, DG75, and CEM cells were either mock-infected or infected with SV40 strains 776-1E, 776-2E, or 776-2E-SM1 and were harvested and analyzed at various d.p.i. Harvest times shown are indicated by the number on each histogram. The cells were stained with anti-CD69-PE, anti-CD80, anti-CD86, or anti-CD25-PE antibodies (BD Pharmingen) and were analyzed by flow cytometry. These results show that SV40 infection affects the expression of lymphocyte surface markers on human B cells by up-regulating CD69 and CD25 and by down-regulating CD80 and CD86. These effects were most pronounced following infections by the miRNA mutant 776-2E-SM1. CEM T cells showed no change in surface expression of CD69, CD80, or CD86 at any day after SV40 infection.

To determine if SV40 infection was associated with changes in the expression levels of cell surface markers on lymphocytes, B cells (DG75, BJAB) and T cells (CEM) were examined during an infection time course. Expression levels of a lymphocyte activation marker, CD69, showed an increase on infected BJAB and DG75 cells (Fig 9A). The most pronounced induction was mediated by the SV40 miRNA-null mutant, 776-2E-SM1. There was no change in CD69 surface levels on infected CEM cells during the time course. Expression of another activation marker, CD25, also was elevated on B cells following infection with the miRNA mutant, but not detectably by the WT strains (Fig 9D). Viral effects on surface expression of the costimulation markers, CD80 and CD86, that play an essential role in generating an antigen-specific immune response were measured. Both CD80 (Fig 9B) and CD86 (Fig 9C) were down-regulated on B cells following infection with the SV40 mutant strain 776-2E-SM1. Virus WT strains 776-1E and 776-2E had little detectable effect on CD80 or CD86 expression on B cells and SV40 infections caused no observable surface changes of those markers on CEM cells.

These observations show that SV40 infection can alter the expression of surface markers on human B cells. The presence of the viral miRNA (encoded by the WT strains) dampens the degree of those viral effects.

## Discussion

This study addressed the nature of the interaction of polyomavirus SV40 with human lymphocytes. We found that B cells and myeloid cells can be infected by SV40. Viral genomes were maintained in established B cell lines (DG75, BJAB, RL) and a myeloid cell line (TF-1a) during several weeks of subculture following infection (Figs 1 and 6). In contrast, the amount of viral DNA retained in an infected T cell line (CEM) steadily decreased, paralleling the calculated loss of input virus by dilutions during cell passage (Fig 1).

SV40 T-ag protein was detected by flow cytometry in a low percentage of infected human B lymphocytes (Fig 2). This T-ag synthesis confirmed expression from the early region of the viral genome in some infected cells. However, T-ag mRNA transcripts were undetectable in the infected B cells under the conditions of our assay. This was probably due to low levels of expression in a fraction of the cells, as very low viral DNA copy numbers (~2) were estimated to be present per infected B cell (Table 1). Similarly, it has been reported that polyomavirus JCV T-ag protein was observed in a low percentage (<10%) of infected human B cells whereas T-ag mRNA was undetectable [13].

Primary lymphocytes from normal donors confirmed the B-cell tropism of SV40 infectivity (Fig 3). Following infection, SV40 viral genomes were detected in primary B cells and monocytes but not in T cells, corroborating the results obtained with the cell lines. Among the normal human donors surveyed, we found that SV40 infections were B-cell tropic in 4 of 6 donors, whereas viral DNA was undetectable in B cells from two other donors. This observation suggests that some individuals may display reduced susceptibility of their lymphocytes to SV40 infection. Future studies involving larger numbers of donors will be necessary to confirm and characterize this possible range in susceptibility. It has been reported that SV40 infection or T-ag transfection can extend the life span of human primary B cells and/or T cells from normal donors [57–60]. Our failure to demonstrate infection of primary T cells may also reflect individual variation in lymphocyte susceptibility to SV40 infection.

Infected B cell lines had the capacity to transmit virus replication potential, as evidenced by increases in SV40 genome copy numbers and expression of early and late SV40 mRNA in recipient susceptible monkey cells (Fig 5). Various virus rescue conditions showed that infectivity could be transferred from infected B cells to permissive TC7 cells by cell lysates, by coculture of live, infected B cells with TC7 cells, and by infectious center assays in which live, infected B cells were placed onto TC7 monolayers and processed under agar for plaque assay (Fig 5; Tables 2 and 3). The underlying mechanism(s) responsible for transfer of infectivity from B cells to permissive cells is unknown, although it is likely that occasional B cells spontaneously produce some infectious virions. It appeared from the infectious center assays that the frequency of SV40 virus rescue from infected B cells was a rare event (~0.01%). It has been described for EBV-immortalized B-lymphoblastoid cell lines that between 1 and 350 viral copies are present per cell and that between 0.1% and 8% of cells may undergo spontaneous reactivation [61,62]. Our data suggest that SV40-infected B cells may produce infectious virus less frequently than seen with EBV, but further studies are necessary to confirm that comparison.

We found that SV40 genetic factors had an effect on virus–lymphocyte interactions. A possible role for SV40 miRNA during infection of lymphocytes was evaluated by comparing the effects of WT SV40 strain 776-2E to those of the mutant derivative 776-2E-SM1. This null mutant lacks the production of the viral miRNA that causes cleavage of early SV40 RNAs, resulting in an increase in large T-ag protein in mutant-infected cells and increased susceptibility of infected cells to lysis by cytotoxic T cells [49]. We previously showed for the first time the *in vivo* expression and function of SV40 miRNA using the Syrian golden hamster model [50]. Animals inoculated with two miRNA negative mutant strains displayed higher tissue viral loads than the parental WT strains, although lymphocytes were not among the tissues examined in that study [50]. In this current study, mutant virus-infected human B lymphocytes and myeloid cells tended to contain higher copy numbers of SV40 DNA over time than did parallel cultures infected with the WT virus (Figs 1 and 6). The miRNA null mutant-infected cells also yielded more infectious virus in rescue experiments long term than the WT-infected cultures (Table 3). Cells infected with mutant 776-2E-SM1 displayed higher percentages of T-ag-expressing cells than those infected with 776-2E or 776-1E and more pronounced changes in expression of cell surface markers, as detected by flow cytometry (Figs 2 and 9). Thus, the enhancement effect of the absence of the viral miRNA on SV40 infections was consistent when addressed using different experimental approaches.

Other polyomaviruses that have been analyzed encode miRNAs, including BKV, JCV, MCPyV, SA12, and MuPyV [63–68]. Also, many viral miRNAs have been identified for herpesviruses. Although diverse in sequence and targets, the viral miRNAs are thought to be involved in several common functions, including regulation of virus gene expression, establishment/maintenance of viral persistent infections, effects on the host cell cycle, and escape from cell killing by the host immune system [67–70]. Human polyomaviruses BKV and MCPyV miRNAs have been found to limit viral replication and/or transcription in host cells and so may facilitate establishment of persistent infections [71,72]. As further evidence of complexity, viral miRNAs appear to target cellular transcripts as well. JCV and BKV miRNAs target cellular ULBP3 expression, a stress-induced ligand that is recognized by a receptor on natural killer (NK) or CD8^+^ T lymphocytes and results in death of the target cell [73,74]. Recently, SV40 has been reported to downregulate expression of ULBP1, another stress-induced ligand, on human cells [75]. Similarly, herpesvirus miRNAs encoded by human cytomegalovirus, Kaposi’s sarcoma-associated herpesvirus (KSHV), and EBV target major histocompatibility complex class I chain-related protein B, another ligand for NK-mediated killing [76,77]. Such targeting of cellular proteins by viral miRNAs could modulate the immune response to virus-infected cells by the host. An intriguing observation is that JCV and BKV miRNAs circulate in blood, urine, and cerebrospinal fluid of patients [78], the biological consequences of which remain to be determined.

Infection with the SV40 miRNA mutant virus in our study appeared to slow the proliferation of some B cells and myeloid cells, as compared to companion cultures infected with SV40 WT strains (Figs 4 and 7), but with minimal concomitant decrease in cell viability (Fig 8). This suggests either that elevated levels of T-ag may be growth inhibitory in lymphocytes or that the viral miRNA may have a cellular target that functions to overcome blocks to cell division induced by SV40 infection. Herpesvirus EBV viral miRNAs have been reported to inhibit apoptosis and promote cell proliferation, with EBV miRNA-negative mutants causing slower cell growth [79,80]. This could serve to increase or stabilize the persistent viral reservoir.

The genomes of virus strains 776-1E and 776-2E contain a single (archetypal, simple) or a double (nonarchetypal, complex) enhancer element in the viral RR, respectively [81]. The viral RR contains the 72-base pair noncoding region with the origin of DNA replication and promoter/enhancer elements that function in regulating viral transcription and replication [82]. A single enhancer is typically present in natural isolates of SV40 or in virus isolated from human tumors whereas duplicated enhancers are usually found in laboratory-adapted strains of SV40 and occasionally in isolates from immunocompromised hosts [30,83,84]. Viruses with complex enhancers have been shown to replicate better in tissue culture [85–87]. In this study, slightly higher levels of SV40 DNA were detected in human B cell lines infected with the 776-2E strain compared to the 776-1E strain (Fig 1). However, any stimulatory effects of a complex RR to yield higher levels of viral DNA replication in lymphocytes were small compared to the effects of lack of viral miRNA. In previous studies, 1E strains of SV40 showed increased tumor incidence in infected hamsters whereas viruses with complex RRs were vertically transmitted from mother to progeny at higher frequency [54,81].

The technique of limiting dilution coupled with qPCR assays revealed that two different infected B cell lines (DG75, BJAB) retained similar low levels of viral DNA (~2 copies per infected cell) even though the percentage of infected cells in the lines differed (Table 1). This suggests that replication of the viral genome may be linked to cell division of infected lymphocytes. Possible mechanisms of DNA viral genome persistence in host cells include episomal maintenance and integration into a host cell chromosome. However, it is known that integration is a very rare event with SV40. Tethering mechanisms have been described for persistent infections in other viral systems by which a viral protein that binds the viral DNA also associates with host cell mitotic chromosomes and mediates the retention and transmission of episomal viral DNA during cell division [88–90]. This mechanism has been described for bovine papillomavirus (BPV), human papillomavirus (HPV), and herpesvirus KSHV in which viral proteins BPV E2, HPV E2, and KSHV LANA are the key viral DNA linkers [91–94]. As SV40 T-ag binds to viral DNA, we postulate that the maintenance of episomal SV40 genomes in lymphoid cells is mediated by binding interactions between T-ag, the viral DNA, and host cell components of mitotic chromosomes. We speculate that tethering by T-ag can occur, even when the expression of T-ag protein in infected lymphocytes is below the level of detection. Future studies are needed to explore this model.

Other polyomaviruses appear to establish persistent infections in B lymphocytes. Human B cell lines or cultures of primary B cells infected with JCV show that a low percentage of cells (1–7%) express viral proteins, maintain viral DNA in 1–2% of cells weeks after infection, and harbor infectious virus recoverable from B cells by inoculation of cell lysates onto permissive cell types [4,8,11,13]. Human polyomaviruses BKV and JCV are known to cause life-long persistent infections affecting most frequently the kidneys, central nervous system, and hematopoietic system [95]. *Ex vivo* data also suggest that human polyomaviruses are lymphotropic. DNA of JCV and BKV have been detected in human tonsils, adenoids, and peripheral blood mononuclear cells [8,10,96,97]. It has been proposed also that human B cells nonproductively infected by JCV could traffic virus across the blood–brain barrier to oligodendrocytes in the brain, initiating the pathology in progressive multifocal leukoencephalopathy [13]. JCV DNA has been detected in primary human B cells from immunocompromised patients at an estimated frequency of 1 viral genome per 20 cell equivalents [6]. Similar to JCV and BKV polyomaviruses in humans, SV40 is not associated with disease in its immunocompetent natural host, the rhesus macaque. However, during coinfection with simian immunodeficiency virus, the widespread host cell range for SV40 becomes apparent with virus detected in multiple tissues, including kidneys, spleen, brain, lymph nodes, lung, and peripheral blood mononuclear cells [28–30]. Thus, a common feature of polyomavirus pathogenesis, exemplified here by SV40, appears to be the establishment of persistent, nonlytic infections in B cells and perhaps myeloid cells. Theoretically, these cells could seed target tissues that support virus replication and result in transmission among hosts. SV40 has been detected in cage waste (excrement, urine) of housed monkeys [98] and in the urine and feces of human adults and children [34,99–103]. It has been proposed that exposure to monkey waste is a source for simian transmission and that a fecal/urine–oral route of transmission occurs among humans [98,102,104].

A potential biological effect of viral infection of lymphocytes is the attenuation of T cell recognition. SV40-infected B cells showed downregulation of two costimulatory molecules, CD80 and CD86 (Fig 9). When expressed by antigen-presenting cells, these molecules provide signals to T cells during antigen-specific priming. In the absence of such signals, the T-cell response is rendered anergic, allowing survival of the antigen-bearing cells. These observations suggest an immune evasion strategy by the virus that would facilitate maintenance of SV40 persistently infected B cells. There could also be more deleterious outcomes, such as cancer development. In the Syrian golden hamster experimental animal model, SV40 can induce tumors of B cell and histiocytic origin [53,105–107]. In humans, SV40 DNA has been detected in B cell lymphomas [39,108–115], expression of the SV40 T-ag protein has been confirmed in some viral DNA-positive lymphoid tumors [110,111,114], and some non-Hodgkins lymphoma patients have been found to have antibodies against SV40 T-ag [116]. There have been other reports which failed to detect SV40 markers in human cancers [39,115]. Suggestions to explain such inconsistencies have included sample selection, assay sensitivity, and laboratory contamination. However, it is likely that the more important factor to consider is the geographic origin of the specimens tested. It has been proposed that SV40 infections in humans were established primarily by exposure to SV40-contaminated oral poliovaccines, which were used in only certain regions [102]. Human infections could be maintained by fecal/urine–oral transmission, predominantly in areas and populations with poor sanitation/living conditions. Infections established in settings with good sanitation would be reduced or eliminated due to interruption of transmission. This model predicts that SV40-positive cancers would generally appear in restricted geographic regions and populations or in individuals recently moved from such areas [102].

In summary, this study showed that SV40 is able to establish persistent infections in human B lymphocytes and myeloid cells. The cells maintain few (about 2) SV40 genome copies per virus-positive cell, with expression of low levels of large T-ag protein detectable in some cells. SV40-infected B cells remain viable and proliferation-competent and display some changes in expression of cell-surface markers. SV40 persistent infections appear to be nonproductive and noncytolytic, but are able to occasionally produce infectious virus. The SV40 miRNA negatively regulates viral replication and viral effects on host lymphocytes. Persistently infected B cells may be an important factor in polyomavirus chronic infections and pathogenesis.
